# Automated volumetric breast density measures: differential change between breasts in women with and without breast cancer

**DOI:** 10.1186/s13058-019-1198-9

**Published:** 2019-10-28

**Authors:** Kathleen R. Brandt, Christopher G. Scott, Diana L. Miglioretti, Matthew R. Jensen, Amir P. Mahmoudzadeh, Carrie Hruska, Lin Ma, Fang Fang Wu, Steven R. Cummings, Aaron D. Norman, Natalie J. Engmann, John A. Shepherd, Stacey J. Winham, Karla Kerlikowske, Celine M. Vachon

**Affiliations:** 1 Department of Radiology, Mayo Clinic, 200 First Street SW, Rochester, MN 55905 USA; 2 Department of Health Sciences Research, Mayo Clinic, 200 First Street SW, Rochester, MN 55905 USA; 3 Kaiser Permanente Washington Health Research Institute, 1730 Minor Avenue, Seattle, WA 98101 USA; 4 Department of Radiology and Biomedical Imaging, University of California, 505 Parnassus Avenue, San Francisco, CA 94143 USA; 5 Division of Research, Kaiser Permanente, 2000 Broadway, Oakland, CA 94612 USA; 6 California Pacific Medical Center Research Institute, 475 Brannan Street #220, San Francisco, CA 94107 USA; 7 Department of Epidemiology and Biostatistics, University of California, 550 16th Street, Second Floor, San Francisco, CA 94158 USA; 8 University of Hawaii Cancer Center, 701 Ilalo Street, Honolulu, HI 96813 USA

**Keywords:** Volumetric density, Breast density, Breast cancer, Tissue asymmetry, Risk

## Abstract

**Background:**

Given that breast cancer and normal dense fibroglandular tissue have similar radiographic attenuation, we examine whether automated volumetric density measures identify a differential change between breasts in women with cancer and compare to healthy controls.

**Methods:**

Eligible cases (*n* = 1160) had unilateral invasive breast cancer and bilateral full-field digital mammograms (FFDMs) at two time points: within 2 months and 1–5 years before diagnosis. Controls (*n* = 2360) were matched to cases on age and date of FFDMs. Dense volume (DV) and volumetric percent density (VPD) for each breast were assessed using Volpara™. Differences in DV and VPD between mammograms (median 3 years apart) were calculated per breast separately for cases and controls and their difference evaluated by using the Wilcoxon signed-rank test. To simulate clinical practice where cancer laterality is unknown, we examined whether the absolute difference between breasts can discriminate cases from controls using area under the ROC curve (AUC) analysis, adjusting for age, BMI, and time.

**Results:**

Among cases, the VPD and DV between mammograms of the cancerous breast decreased to a lesser degree (− 0.26% and − 2.10 cm^3^) than the normal breast (− 0.39% and − 2.74 cm^3^) for a difference of 0.13% (*p* value < 0.001) and 0.63 cm^3^ (*p* = 0.002), respectively. Among controls, the differences between breasts were nearly identical for VPD (− 0.02 [*p* = 0.92]) and DV (0.05 [*p* = 0.77]). The AUC for discriminating cases from controls using absolute difference between breasts was 0.54 (95% CI 0.52, 0.56) for VPD and 0.56 (95% CI, 0.54, 0.58) for DV.

**Conclusion:**

There is a small relative increase in volumetric density measures over time in the breast with cancer which is not found in the normal breast. However, the magnitude of this difference is small, and this measure alone does not appear to be a good discriminator between women with and without breast cancer.

## Background

Mammographic breast density (MBD) has been shown to be a major risk factor for breast cancer [1–3] and inversely related to the sensitivity of mammography [4, 5]. The breast density measure most widely used in clinical practice is the Breast Imaging Reporting and Data System (BI-RADS) [6], which consists of four categories of increasing density reflecting the risk of tumor masking and breast cancer [7, 8]. However, it is a subjective [9–11] woman-level assessment of overall density determined by the interpreting radiologist.

Automated volumetric density measures for full-field digital mammography (FFDM) have the potential to replace or at least complement the radiologist’s BI-RADS density assessment as they provide objective, reproducible density estimates [12, 13]. Automated density measures have shown correlation with clinical BI-RADS density categories, similar positive association with breast cancer risk, and ability to stratify screening outcomes [7, 8, 14, 15]. In addition to assigning an overall woman-level BI-RADS-like density category, automated systems also calculate total breast volume, dense volume (DV), and volumetric percent density (VPD) for each breast separately. This study investigates the potential for the automated density measures calculated for each breast separately to detect potentially relevant unilateral breast changes in density over time.

Breast cancer usually develops in one breast and has the same radiographic X-ray attenuation as normal dense fibroglandular tissue [2, 16–20]. If unilateral cancer is present, density from the cancer itself may result in asymmetric increases in density calculations in the affected breast. Prior studies have found mammographic density or feature asymmetry between breasts on mammograms prior to diagnosis to be predictive of individual near-term breast cancer risk using complex computerized feature analysis [21–23] or computer-aided software [24]. In this retrospective study, we analyze the commercially available automated Volpara™ VPD and DV outputs for each breast separately to examine whether longitudinal changes in the breast with cancer, up to the time of diagnosis, differ from the contralateral normal breast. We hypothesize that women with breast cancer will have an asymmetric density increase in the affected breast compared to the unaffected breast. We also evaluate these changes in a set of women without breast cancer and compare the changes in women with cancer to these healthy controls. Together, these studies address whether automated volumetric breast density measures can detect clinically important asymmetric density changes over time between breasts affected and not affected by breast cancer.

## Methods

### Study population

The participating studies included two retrospective case-control studies nested within large breast screening facilities at Mayo Clinic Rochester (MCR) and in the Bay Area that participate in the San Francisco Mammography Registry (SFMR) [8]. Passive permission was obtained from women at SFMR facilities; at Mayo Clinic, a general research authorization was obtained allowing for retrospective chart reviews. Each study was approved by relevant institutional review boards and was HIPPA compliant.

#### SFMR

Since 2006, women were included in the underlying mammography cohort if they had a FFDM at one of four facilities [8]. Women were eligible as cases if they had a unilateral invasive incident breast cancer reported to the California Cancer Registry from January 2007 to May 2014 (*n* = 1322) and did not have breast implants or a prior breast cancer diagnosis (DCIS or invasive).

#### MCR

Women from the tri-state region of Minnesota, Iowa, and Wisconsin who had FFDM at MCR between April 2008 and December 2015 serve as the underlying mammography cohort [8]. Women were eligible as cases if they had a unilateral invasive incident breast cancer reported to the Mayo Clinic Tumor Registry through December 2015 (*n* = 648) and did not have breast implants or a prior breast cancer diagnosis (DCIS or invasive).

Because our goal was to assess the density change between breasts over time, we required a bilateral screening or diagnostic FFDM within 2 months prior to the cancer diagnosis and at least one additional FFDM 1–5 years prior to diagnosis to be included as cases. Patient age, clinical risk factors, and tumor characteristics were obtained from clinical questionnaires or abstracted from medical records and tumor registries. Due to missing data on menopause, we defined menopausal status as age 55 or older [1, 25].

Women without prior breast cancer, and with bilateral FFDM over the same time period, were matched to each case on age (within 5 years), race (as close as feasible), date of mammogram (within 1 year), FFDM machine (exact), and facility (exact).

### Volumetric density measures

Volpara™ [26] is a fully automated method for assessing volumetric breast density that uses the measured breast thickness and X-ray attenuation in the raw or “for processing” image for each breast to create estimates of dense and non-dense tissue volume for each pixel. Summing the dense pixel volumes provides total DV. Dividing DV by total breast volume and multiplying by 100 defines VPD. For each breast, the final DV and VPD, an average of the cranio-caudal and mediolateral oblique density estimates, were analyzed. The clinical BI-RADS four-category tissue composition assessment used in this study was obtained from the earliest mammogram. Over this period, the fourth edition of the American College of Radiology BI-RADS was used, classifying the breast density into one of four categories: entirely fat, scattered fibroglandular densities, heterogeneously dense, and extremely dense [27].

### Statistical methods

Baseline characteristics were summarized using number and percentage for categorical variables and median and quartiles for continuous variables. Using the Volpara™ calculations for each breast, the change in DV and VPD between mammograms was calculated for each breast separately. To assess the difference between breasts among cases, we subtracted the change in DV and VPD in the normal contralateral breast from the change in the breast with cancer (ipsilateral). The difference (ipsilateral change minus contralateral change) was examined by tumor size, clinical BI-RADS density (grouped into dense (heterogeneously or extremely dense) vs. non-dense (fatty or scattered density)), and menopausal status (defined as age ≥ 55 years) at the time of the earliest mammogram. The above analysis was done in a similar fashion in the controls by defining the “ipsilateral” and “contralateral” sides based on the sides of the matched case. By analyzing the difference in the change between the two breasts within each woman, we control for systemic variables that should affect both breasts equally, such as aging, hormone therapy, and weight change. Statistical significance of the change was evaluated using the Wilcoxon signed-rank test.

A secondary analysis was performed to simulate clinical practice, where the cancerous breast is not known at the time of the mammogram review. In this analysis, the change in VPD and DV between mammograms was again defined for left and right breasts separately. However, the absolute difference in the change between breasts was calculated for cases and controls without knowledge of the cancerous breast; change was defined as the absolute value of the maximum difference between the two breasts. The correlation of change between the two breasts was examined using Lin’s concordance correlation coefficients, which summarizes the agreement along the line of identity. The cumulative distribution of the absolute difference in VPD and DV was examined in cases and controls. Logistic regression was used to evaluate the ability of the absolute difference to discriminate breast cancer case status from controls, with and without adjustment for age, BMI, change in BMI, and time between mammograms. The difference in the area under the ROC curve (AUC) between the baseline model (age, BMI, BMI changes, and time between mammograms) and the model including the absolute difference was calculated and summarized with corresponding 95% confidence interval. The analysis was done using SAS version 9.4 (Cary, NC), and statistical significance was set at *p* ≤ 0.05.

## Results

A total 1160 of the 1970 potential cancer cases met eligibility criteria. Reasons for exclusion included 795 without an available bilateral mammogram within 2 months of diagnosis and 15 without a prior bilateral mammogram within 5 years of diagnosis. Distributions were not markedly different when analyzed by study cohort (Additional file 1: Table S1); therefore, combined results are presented. The median age at diagnosis was 61 years. A total of 28% of cases had a family history of breast cancer in first-degree relatives and 31% were pre-menopausal. Of the 88% (1020/1160) of women with a recorded clinical BI-RADS density at the earliest mammogram, 53% were non-dense and 47% were dense. A total of 76% of cancers were ≤ 2 cm, 21% were 2.1–5 cm, and 3% were> 5 cm. A total of 21 (2%) tumors had no recorded size. Family history of breast cancer and breast density measures differed by case and control status while age, menopausal status, and BMI were similar (Table 1).

**Table 1:** Risk factors and clinical characteristics of cases and controls. Cases are also shown stratified by tumor size

	Overall cases (*N* = 1160)	Tumor size			
		≤ 2 cm (*N* = 860)	2.1–5 cm (*N* = 243)	> 5 cm (*N* = 36)	Controls (*N* = 2360)
Age, median (Q1, Q3)*	61.0 (53, 70)	61.1 (53, 71)	60.0 (52, 69)	57.7 (50, 67)	61.4 (54, 70)
Body mass index, median (Q1, Q3)*	25.8 (22.7, 30.5)	25.8 (22.8, 30.3)	26.4 (22.5, 30.8)	28.6 (24.4, 32.9)	25.8 (22.9, 30.2)
Race					
White	943 (81.3%)	706 (82.1%)	193 (79.4%)	32 (88.9%)	2028 (85.9%)
Asian	139 (12.0%)	95 (11.0%)	37 (15.2%)	2 (5.6%)	203 (8.6%)
African-American	26 (2.2%)	18 (2.1%)	6 (2.5%)	0 (0.0%)	39 (1.7%)
Hispanic	21 (1.8%)	17 (2.0%)	3 (1.2%)	0 (0.0%)	43 (1.8%)
Others/mixed	31 (2.7%)	24 (2.8%)	4 (1.6%)	2 (5.6%)	47 (2.0%)
Interval between mammograms, median (Q1, Q3)	3.0 (1.9, 3.9)	3.0 (1.9, 4.0)	3.0 (1.9, 3.9)	2.9 (1.6, 3.9)	3.1 (2.0, 4.1)
First degree family Hx B.C.*	317 (27.6%)	247 (29.1%)	59 (24.5%)	6 (16.7%)	479 (20.4%)
Post-menopausal* (age 55+)	805 (69.4%)	605 (70.3%)	161 (66.3%)	21 (58.3%)	1675 (71.0%)
Screening mammogram within 2 months of diagnosis	1043 (90%)	784 (91%)	212 (87%)	27 (75%)	–
BI-RADS density*					
a	132 (12.9%)	107 (14.1%)	20 (9.5%)	3 (10.0%)	463 (20.5%)
b	408 (40.0%)	309 (40.6%)	81 (38.6%)	9 (30.0%)	973 (43.1%)
c	391 (38.3%)	275 (36.1%)	94 (44.8%)	17 (56.7%)	684 (30.3%)
d	89 (8.7%)	70 (9.2%)	15 (7.1%)	1 (3.3%)	138 (6.1%)
VPD (%)*					
Median	6.9	6.6	8.1	9.0	5.9
Q1, Q3	4.8, 11.3	4.6, 10.8	5.2, 12.9	5.7, 12.8	4.3, 9.7
Range	(2.3–36.0)	(2.3–36.0)	(2.4–29.7)	(3.3–24.8)	(2.0–36.3)
DV (cm^3^)*					
Median	56.0	53.5	62.6	81.6	47.7
Q1, Q3	41.5, 79.4	40.0, 73.5	45.1, 90.4	62.2, 110.7	35.9, 64.8
Range	(12.4–281.1)	(12.4–265.4)	(17.7–212.6)	(22.2–199.2)	(10.8–314.8)

Twenty-one case subjects with unknown tumor size, 50 case subjects with unknown BMI, 13 case subjects with unknown family history, 140 subjects with unknown BI-RADS density

*BI-RADS* Breast Imaging Reporting and Data System 4th edition, *VPD* volumetric percent density, *DV* dense volume

*Determined at the time of the earliest mammogram

The median (IQR) time between the oldest prior mammogram within 5 years and the mammogram closest to the diagnosis was 3.0 years (IQR 1.9, 3.9). Over this period, both VPD and DV decreased on average among both cases and controls (Table 2). Among cases, the VPD and DV of the breast with cancer decreased to a lesser degree than the breast without cancer (Table 2; Additional file 1: Table S2). For cases overall, the cancerous (ipsilateral) breast VPD decreased 0.26% and the contralateral breast VPD decreased 0.39% for a difference of 0.13% (*p* value < 0.001). For DV, the ipsilateral breast decreased 2.10 cm^3^ and the contralateral breast decreased 2.74 cm^3^ for a difference of 0.63 cm^3^ (*p* = 0.002).

**Table 2:** Median (interquartile range) changes in volumetric density measures by breast side and differences between sides, cases, and controls, overall and stratified on BI-RADS density

	Cases				Controls			
	Ipsilateral change*	Contralateral change*	Difference	*p* value	Ipsilateral change*	Contralateral change*	Difference	*p* value
Overall (*n* = 1160 cases/2360 controls)								
VPD (%)	− 0.26 (− 1.34, 0.70)	− 0.39 (− 1.39, 0.39)	0.13 (− 0.69, 1.06)	< .001	− 0.29 (− 1.26, 0.48)	− 0.28 (− 1.35, 0.50)	− 0.02 (− 0.72, 0.79)	0.92
DV (cm^3^)	− 2.10 (− 9.65, 5.06)	− 2.74 (− 10.25, 3.34)	0.63 (− 6.14, 8.80)	0.002	− 1.82 (− 8.09, 3.60)	− 1.89 (− 8.49, 3.84)	0.05 (− 5.75, 6.07)	0.77
Non-dense (*n* = 540 cases/1436 controls)								
VPD (%)	− 0.11 (− 0.76, 0.66)	− 0.23 (− 0.86, 0.33)	0.11 (− 0.49, 0.83)	< .001	− 0.21 (− 0.85, 0.43)	− 0.18 (− 0.89, 0.40)	− 0.02 (− 0.60, 0.66)	0.93
DV (cm^3^)	− 1.28 (− 7.21, 4.98)	− 1.73 (− 8.13, 3.17)	0.63 (− 4.88, 8.80)	0.002	− 1.66 (− 7.01, 3.09)	− 1.58 (− 7.36, 3.17)	0.09 (− 5.59, 5.69)	0.89
Dense breast (*n* = 480 cases/822 controls)								
VPD (%)	− 0.65 (− 2.49, 0.82)	− 0.91 (− 2.52, 0.56)	0.21 (− 1.01, 1.28)	0.11	− 0.65 (− 2.33, 0.81)	− 0.79 (− 2.33, 0.82)	0.00 (− 1.15, 1.13)	0.97
DV (cm^3^)	− 3.42 (− 13.23, 4.34)	− 4.30 (− 13.59, 3.33)	0.61 (− 7.62, 8.35)	0.38	− 2.53 (− 10.01, 4.77)	− 2.72 (− 10.93, 5.12)	− 0.02 (− 6.16, 6.83)	0.77

Control sides were defined as the same as matched cases. Determined at the time of the earliest mammogram

*BI-RADS* Breast Imaging Reporting and Data System, *VPD* volumetric percent density, *DV* dense volume

*Change was calculated by subtracting the density at the earliest mammogram from the density at the mammogram closest to diagnosis. A negative value reflects a decrease over time

When stratified by BI-RADS density (dense vs. non-dense) assessed at the earliest mammogram, tumor size and menopausal status, the VPD and DV of the cancerous ipsilateral breast decreased to a lesser degree than the contralateral breast without cancer in all subsets; but not all results reached statistical significance. Women with non-dense breasts (*n* = 540) had a difference of 0.11% (*p* < 0.001) and 0.63 cm^3^ (*p* = 0.002) between the breast with cancer and the normal breast for VPD and DV, respectively. Women with dense breasts (*n* = 480) had a difference of 0.21% (*p* = 0.11) for VPD and 0.61 cm^3^ (*p* = 0.38) for DV (Table 2). The difference in density change between breasts was accentuated with increasing tumor size, especially for DV (Table 3). Women with cancer ≤ 2-cm had a VPD difference of 0.07% (*p* = 0.23) and DV difference of 0.02 cm^3^ (*p* = 0.76) between the cancerous and non-cancerous breasts. For women with cancers 2.1–5 cm, the difference increased to 0.40% (*p* < 0.001) for VPD and 4.15 cm^3^ (*p* < 0.001) for DV. For women with cancers > 5 cm, the difference was even greater with 1.18% for VPD (*p* < 0.001) and 12.97 cm^3^ (*p* < 0.001) for DV. Similar results were found for post-menopausal women (age ≥ 55) when analyzed as a sub-group (Additional file 1: Tables S3 and S4).

**Table 3:** Median (interquartile range) changes in volumetric density measures by breast side and differences between sides for breast cancer cases, stratified on tumor size

	Ipsilateral change**	Contralateral change**	Difference	*p* value
Tumors ≤ 2 cm (*N* = 860)				
VPD (%)	− 0.30 (− 1.24, 0.65)	− 0.32 (− 1.26, 0.47)	0.07 (− 0.74, 0.87)	0.23
DV (cm^3^)	− 2.52 (− 9.70, 3.98)	− 2.72 (− 9.51, 3.35)	0.02 (− 6.62, 7.03)	0.76
Tumors 2–5 cm (*N* = 243)				
VPD (%)	− 0.18 (− 1.67, 0.93)	− 0.67 (− 2.00, 0.20)	0.40 (− 0.66, 1.63)	< .001
DV (cm^3^)	− 0.93 (− 10.04, 9.64)	− 3.03 (− 12.97, 3.49)	4.15 (− 4.83, 13.09)	< .001
Tumors > 5 cm (*N* = 36)				
VPD (%)	0.34 (− 1.27, 2.13)	− 0.45 (− 1.74, 0.04)	1.18 (0.02, 2.47)	< .001
DV (cm^3^)	11.78 (− 0.99, 25.14)	− 3.12 (− 9.53, 1.97)	12.97 (2.72, 24.89)	< .001

*VPD* volumetric percent density, *DV* dense volume

**Change was calculated by subtracting the density at the earliest mammogram from the density at the mammogram closest to diagnosis. A negative value reflects a decrease over time

We also assessed the bilateral density changes over time from a set of controls (*N* = 2360), selecting the sides corresponding to the matched case. Similar to breast cancer cases, mammograms spanned a median of 3.1 years (IQR 2.0. 4.1), and density decreased on average over this time period (Table 2). Unlike the cases, among controls, the differences in breast density measures between breasts were nearly identical over time with a difference of − 0.02% (*p* = 0.92) for VPD and 0.05 cm^3^ (*p* = 0.77) for DV (Table 2). Results were virtually identical when stratifying by BI-RADS density: there was little difference over time in VPD and DV for controls with non-dense breasts (difference − 0.02% (*p* = 0.93) and 0.09 cm^3^ (*p* = 0.89), respectively) and for those with dense breasts (difference − 0.00% (*p* = 0.97) and − 0.02 cm^3^ (*p* = 0.77), respectively). Similar findings were seen among the subset of post-menopausal women (age ≥ 55) (Additional file 1: Table S3).

**Fig. 1:**
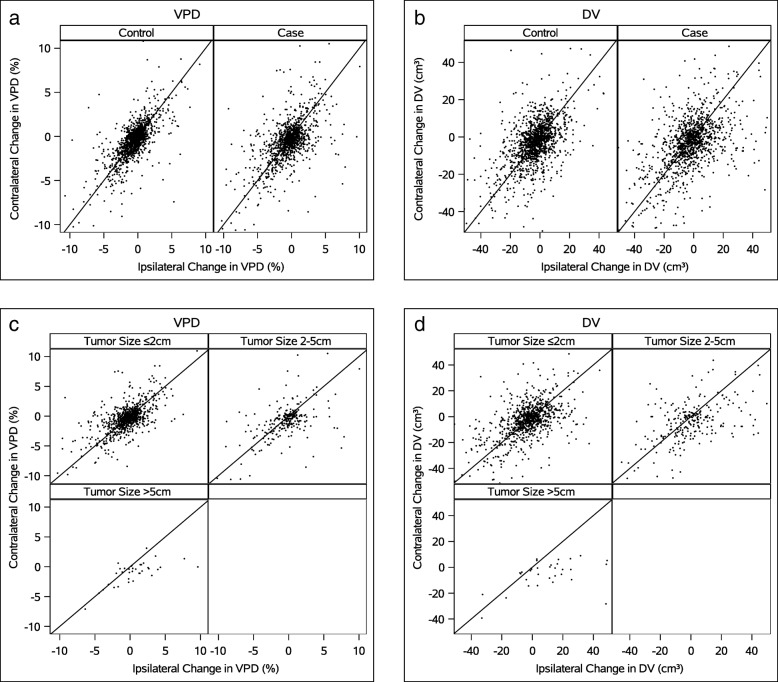
**a**–**d** Scatter plots of ipsilateral vs. contralateral changes for cases, or matched side for controls, in volumetric percent density (VPD) (**a**), dense volume (DV) (**b**), by tumor size in cases only VPD (**c**) and DV (**d**). Ipsilateral change is on the *x*-axis, and contralateral change is on the *y*-axis. The 45° line represents equal changes in both breasts. Subjects with a smaller decrease in density of the ipsilateral breast relative to contralateral are below the 45° line. *Correlation coefficients: **a** cases VPD = 0.57 (0.43, 0.61), controls VPD = 0.72 (0.70, 0.75); **b** cases DV = 0.48 (0.44, 0.53), DV controls = 0.62 (0.59, 0.65); **c** cases VPD <= 2 cm = 0.61 (0.56, 0.65), 2–5 cm = 0.50 (0.40, 0.58), and > 5 cm = 0.51 (0.31, 0.67); **d** cases DV <= 2 cm = 0.52 (0.46, 0.56), 2–5 cm = 0.48 (0.39, 0.57), and > 5 cm = 0.15 (0.01, 0.30)

Overall, among cases and controls, right and left breast VPD and DV differences over time were strongly correlated (Fig. 1a, b). However, for breast cancer cases, the correlation between right and left breast differences in VPD and DV was less strong with increasing tumor size (Fig. 1c, d).

**Fig. 2:**
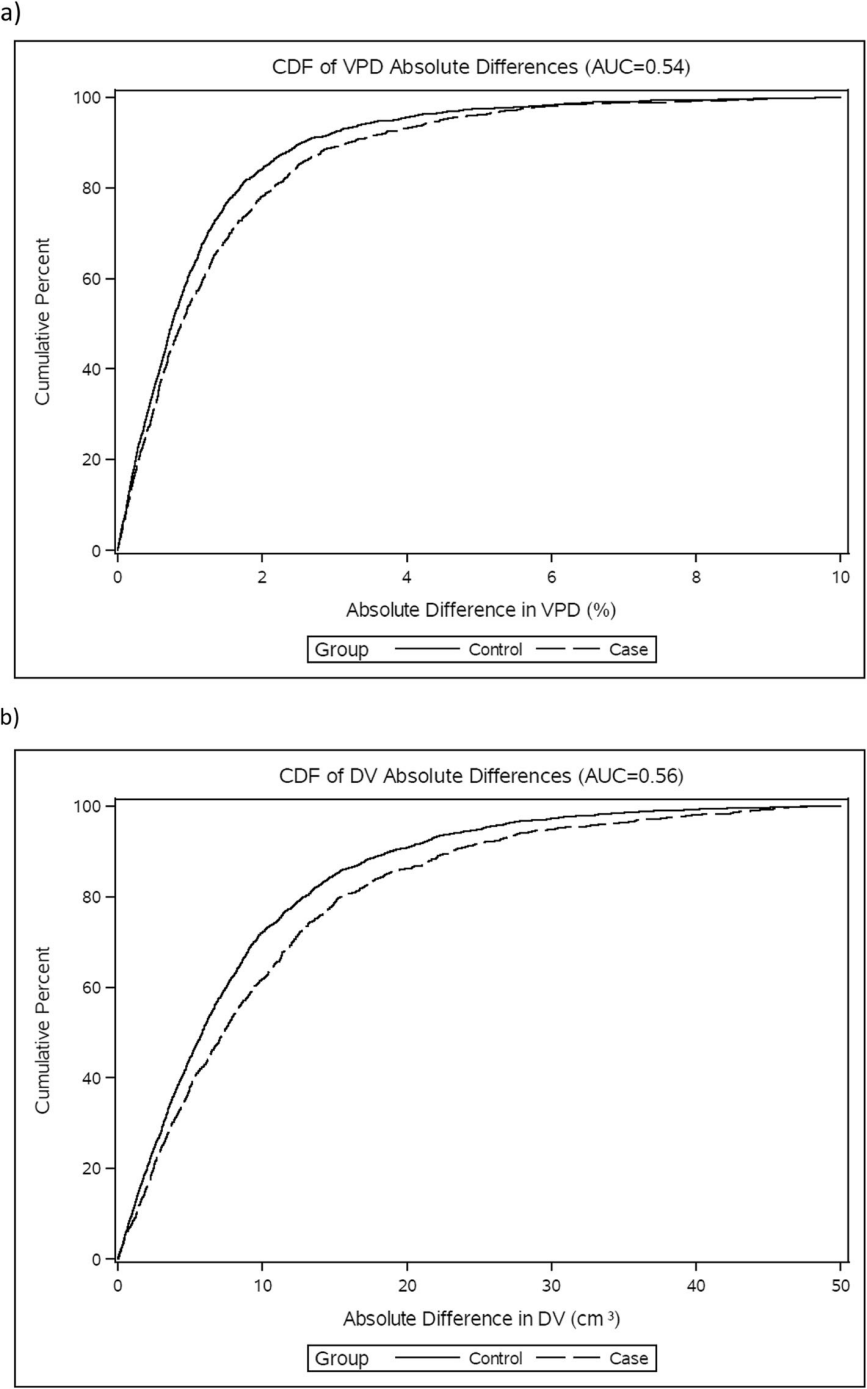
Cumulative distribution function (CDF) of absolute difference in volumetric percent density (VPD) (**a**) and dense volume (DV) (**b**) for cases and controls

In order to simulate clinical practice, where the laterality of malignancy is not known at the time of mammogram interpretation, we compared the absolute difference in density change between breasts among cases and controls. The cumulative distribution of this difference for cases and controls is shown in Fig. 2a, b; in general, differences in controls were smaller than that in cases. Differences of VPD of less than 2% were seen in 84% of controls vs. 78% of cases. Differences in DV of less than 10 cm^3^ were observed in 72% of controls vs. 61% of cases. The AUCs corresponding to the models for discriminating cases from controls using absolute difference between breasts (and including age, BMI, change in BMI, and time between mammograms) in VPD and DV were 0.54 (95% CI 0.52, 0.56) and 0.56 (95% CI 0.54, 0.58), respectively. Importantly, the asymmetry measures only slightly improved discrimination over the baseline model (AUC for discriminating cases and controls using the difference increased by 0.02 (95% CI 0.01, 0.03) for VPD and 0.03 (95% CI 0.02, 0.05) for DV.

## Discussion

Commercially available automated density assessment methods provide volumetric density calculations for each breast separately in addition to assigning a single overall density category per woman, similar to clinical BI-RADS. We evaluated the potential of Volpara™-automated density measures (VPD and DV) calculated for each breast separately to identify clinically relevant differential change between breasts in women with breast cancer. Comparing the automated measures at the time of diagnosis to those from prior mammograms within 5 years, we found that VPD and DV decreased on average in both breasts, but to a lesser degree in the breast with cancer. Density measures also decreased over time in the controls, but this decrease was symmetric with no significant difference between breasts.

Prior studies report that mammographic feature asymmetry between breasts, using complex computerized feature analysis, predicts individual near-term breast cancer risk [21–23, 28] on the next sequential screening mammogram. Ericksonn et al. found differences between breasts in density, and the number of computer-detected microcalcifications and masses was associated with short-term risk of being diagnosed with breast cancer [24]. Only one study by Tan et al. [28] analyzed the longitudinal change between mammograms as we did in this study. Unlike prior studies, we evaluated a commercially available automated density system and included the mammogram at the time of diagnosis. We compared the difference in VPD and DV over time in each breast separately for cases and controls utilizing the values that are included in the clinical Volpara™ report. Our results showing that volumetric breast density measures decreased over time, even for women older than 55, considered post-menopausal for this analysis, are consistent with other published studies [3, 29, 30]. In addition, we found that right and left breast density decreased symmetrically for most women. In cases, we did find that VPD and DV decreased less over time in the breast with cancer than in the normal breast. However, this differential change between breasts is often small and did not reach statistical significance for cases with cancers less than or equal to 2 cm or with dense breasts. It does not appear that these differences reach a threshold where they can provide clinically useful information. This finding is best illustrated by the scatter plots of density change by breast laterality where there were a similar number of cases and controls that show asymmetric change. Even in the subset of women with the largest tumors (> 5 cm) which show the greatest differential changes, we note some controls with differential changes of similar magnitude, limiting clinical significance.

The attenuated decrease in density in the breast with cancer, compared to the normal breast, found in this study is consistent with our hypothesis that the presence of breast cancer can result in an asymmetric density increase in that breast. However, this finding alone does not appear to be a good discriminator between cases and controls using the automated density method in this study. Using the absolute difference in DV and VPD between the two breasts, irrespective of knowing which breast has cancer, the improvement in the discrimination of cases from controls was minimal (change in AUC was 0.02 and 0.03 for VPD and DV, respectively). This automated density measure may incrementally improve discrimination, but our results suggest that it cannot be used in a stand-alone fashion in clinical practice to determine whether further examination is indicated.

Our findings in controls provide important information for future studies. Many published studies evaluating breast density and cancer risk use the non-cancerous breast to avoid possible bias in the density measurement [14, 31, 32]. The symmetric density change over time in our large control group indicates that the small asymmetric change detected between breasts in women with breast cancer is likely real and not “noise” in the system. However, the contribution of cancer itself to DV and VPD appears minimal, even for the largest tumors, and this magnitude of difference is not likely to significantly alter density measures used to estimate the chance of masking effect or the risk of developing breast cancer. This has implications for breast density research studies that only have a unilateral mammogram available at the time of diagnosis.

The strengths of this study include the large number of cases and controls from two breast imaging practices using FFDM. In addition, by comparing right and left breasts, we controlled for variation in density due to systemic factors that affect both breasts such as weight change, hormones, and aging. However, by restricting our study to participants with a bilateral mammogram near diagnosis, we eliminated multiple women and potentially reduced the generalizability of our findings. The relatively small number of cases with tumors greater than 2 cm at diagnosis limited our ability to perform subset analyses. We also evaluated FFDM raw images rather than tomosynthesis, which is rapidly replacing FFDM in clinical practice. Although studies have shown a high correlation between FFDM and tomosynthesis density measures assessed using Volpara™ [33, 34], it is unknown if the asymmetric change in the density in the breast with cancer found in this study will be reproduced, or perhaps more pronounced, with tomosynthesis density assessment. We acknowledge that accurate assessment of volumetric density change requires similar mammogram views and acquisition over the time period. Improving the co-registration of images may provide better measures of density change and asymmetry that will be more strongly associated with the presence of breast cancer. Further, as automated density systems mature, the ability to detect subtle changes in density may improve.

## Conclusion

The results of this study showed a relative increase of DV and VPD over time in the breast with cancer compared to the contralateral normal breast, as well as compared to a set of healthy controls. This automated asymmetry density measure may incrementally improve discrimination of cancer risk, but our results suggest that it cannot be used in a stand-alone fashion in clinical practice to determine whether further examination is indicated.

## Supplementary information

**Additional file 1.** Supplementary tables.

## Data Availability

The datasets generated and/or analyzed during the current study would be available with the appropriate permissions, including collaborative arrangements, an application process, and appropriate data transfer agreements.

## Abbreviations

MBD: Mammographic breast density
BI-RADS: Breast Imaging Reporting and Data System
FFDM: Full-field digital mammography
DV: Dense volume
VPD: Volumetric percent density
CC: Cranio-caudal
MLO: Mediolateral oblique
