# Supplementary material for: Automated volumetric breast density measures: differential change between breasts in women with and without breast cancer
Source: Breast Cancer Res. 2019 Oct 28;21:118. doi: 10.1186/s13058-019-1198-9 (PMC6819393; doi:10.1186/s13058-019-1198-9)
Supplement: Supplementary file 1 — Additional file 1. Supplementary tables. [file 13058_2019_1198_MOESM1_ESM.docx]

**Table S1: Risk Factor Distribution for Cases and Controls, stratified by study.**

|  |  | | |  |
| --- | --- | --- | --- | --- |
|  | **MCR**  **Cases** | **MCR**  **Controls** | **SFMR**  **Cases** | **SFMR**  **Controls** |
|  | (N=498) | (N=1389) | (N=662) | (N=971) |
| **Age, median (Q1, Q3)*** | 62.1 (54, 70) | 61.7 (54, 70) | 60.0 (52, 70) | 61.0 (53, 70) |
|  |  |  |  |  |
| **Body mass index median (Q1, Q3)) *** | 27.8 (24.2, 32.8) | 27.6 (24.2, 32.2) | 24.5 (21.9, 28.3) | 24.0 (21.6, 27.4) |
|  |  |  |  |  |
| **Race** |  |  |  |  |
| White | 485 (97.4%) | 1355 (97.6%) | 458 (69.2%) | 673 (69.3%) |
| Asian | 6 (1.2%) | 11 (0.8%) | 133 (20.1%) | 192 (19.8%) |
| African American | 1 (0.2%) | 0 (0.0%) | 25 (3.8%) | 39 (4.0%) |
| Hispanic | 3 (0.6%) | 8 (0.6%) | 18 (2.7%) | 35 (3.6%) |
| Other/mixed | 3 (0.6%) | 15 (1.1%) | 28 (4.2%) | 32 (3.3%) |
|  |  |  |  |  |
| **Interval between mammograms**, median (Q1, Q3) | 3.1 (2.0, 4.2) | 3.3 (2.0, 4.2) | 2.7 (1.7, 3.4) | 2.7 (1.7, 3.6) |
|  |  |  |  |  |
| **1^st^ degree family Hx B.C. *** | 142 (29.1%) | 299 (21.6%) | 175 (26.6%) | 180 (18.5%) |
|  |  |  |  |  |
| **Post-menopausal *** | 356 (71.5%) | 982 (70.7%) | 449 (67.8%) | 693 (71.4%) |
| (age 55+) |  |  |  |  |
|  |  |  |  |  |
|  |  |  |  |  |
| **BI-RADS*** |  |  |  |  |
| a | 88 (17.7%) | 336 (24.2%) | 44 (8.4%) | 127 (14.6%) |
| b | 201 (40.4%) | 615 (44.3%) | 207 (39.7%) | 358 (41.2%) |
| c | 181 (36.3%) | 373 (26.9%) | 210 (40.2%) | 311 (35.8%) |
| d | 28 (5.6%) | 65 (4.7%) | 61 (11.7%) | 73 (8.4%) |
|  |  |  |  |  |
| **VPD (%) *** |  |  |  |  |
| Median | 5.7 | 5.3 | 8.2 | 7.3 |
| Q1, Q3 | 4.5, 9.0 | 4.1, 8.3 | 5.3, 13.3 | 4.8, 11.9 |

**Table S1: Risk Factor Distribution for Cases and Controls (Continued)**

|  |  | | |  |
| --- | --- | --- | --- | --- |
|  | **MCR**  **Cases** | **MCR**  **Controls** | **SFMR**  **Cases** | **SFMR**  **Controls** |
|  | (N=498) | (N=1389) | (N=662) | (N=971) |
| Range | (2.6-28.7) | (2.2-35.0) | (2.3-36.0) | (2.0-36.3) |
|  |  |  |  |  |
| **DV (cm^3^) *** |  |  |  |  |
| Median | 57.4 | 48.2 | 54.5 | 46.5 |
| Q1, Q3 | 43.4, 78.6 | 37.4, 63.5 | 40.0, 79.8 | 34.3, 67.0 |
| Range | (19.4-199.2) | (12.4-208.0) | (12.4-281.1) | (10.8-314.8) |
| * Determined on earliest mammogram.  21 case subjects with unknown tumor size, 50 case subjects with unknown BMI, 13 case subjects with unknown family history, 140 subjects with unknown BIRADS density. | | | | |

| **Table S2: Median (Interquartile Range) Volumetric Density Measures at each time point by Breast Side, Cases and Controls.** | | | | | | | | |
| --- | --- | --- | --- | --- | --- | --- | --- | --- |
| **Cases (N=1160)** | | | | | **Controls (N=2360)** | | | |
|  | **First time point** | **Second time point** | **Change** |  | **First time point** | **Second time point** | **Change** |  |
| **Ipsilateral**  **VPD (%)** | 7.4  (4.9, 12.3) | 7.1  (4.9, 11.2) | -0.26  (-1.34, 0.70) |  | 6.3  (4.5, 10.5) | 5.9  (4.3, 9.8) | -0.29  (-1.26, 0.48) |  |
| **Contralateral VPD (%)** | 7.4  (4.9, 12.1) | 6.9  (4.6, 11.4) | -0.39  (-1.39, 0.39) |  | 6.4  (4.4, 10.7) | 6.0  (4.3, 9.8) | -0.28  (-1.35, 0.50) |  |
| **Ipsilateral**  **DV (cm^3^)** | 58.6  (42.8, 83.2) | 56.7  (41.6, 80.6) | -2.10  (-9.65, 5.06) |  | 50.2  (37.4, 68.8) | 48.0  (35.8, 64.5) | -1.82  (-8.09, 3.60) |  |
| **Contralateral**  **DV (cm^3^)** | 58.2  (41.7, 82.9) | 54.2  (39.7, 77.5) | -2.74  (-10.25, 3.34) |  | 50.1  (37.4, 70.4) | 47.9  (35.8, 66.3) | -1.89  (-8.49, 3.84) |  |

**Table S3: Median (IQR) Changes and Differences Between Sides, Cases and Controls Age 55+, Overall and Stratified on Density**

| **Cases** | | | | | **Controls** | | | |
| --- | --- | --- | --- | --- | --- | --- | --- | --- |
|  | **IPSI Change** | **Contra Change** | **Difference** | **P-Value** | **IPSI Change** | **Contra Change** | **Difference** | **P-Value** |
| **Overall (n=805 Cases/1675 Controls)** | | | | | | | | |
| **VPD (%)** | -0.14  (-0.95, 0.70) | -0.27  (-1.18, 0.39) | 0.12  (-0.61, 0.95) | <.001 | -0.21  (-0.96, 0.51) | -0.17  (-0.99, 0.57) | -0.02  (-0.67, 0.72) | 0.79 |
| **DV (cm^3^)** | -1.53  (-7.49, 4.34) | -2.08  (-8.62, 3.02) | 0.80  (-4.95, 8.67) | <.001 | -1.59  (-7.06, 3.41) | -1.58  (-7.25, 3.37) | 0.03  (-5.51, 5.77) | 0.82 |
|  |  |  |  |  |  |  |  |  |
| **BI-RADS 1/2 (n=449 Cases/1149 Controls)** | | | | | | | | |
| **VPD (%)** | -0.10  (-0.69, 0.64) | -0.21  (-0.79, 0.35) | 0.09  (-0.50, 0.82) | 0.005 | -0.17  (-0.76, 0.46) | -0.14  (-0.78, 0.43) | -0.02  (-0.58, 0.65) | 0.86 |
| **DV (cm^3^)** | -1.12  (-6.47, 4.54) | -1.25  (-7.38, 3.06) | 0.49  (-4.72, 8.14) | 0.01 | -1.36  (-6.45, 3.12) | -1.48  (-6.74, 2.85) | 0.08  (-5.44, 5.67) | 0.77 |
|  |  |  |  |  |  |  |  |  |
| **BI-RADS 3/4 (n=269 Cases/464 Controls)** | | | | | | | | |
| **VPD (%)** | -0.47  (-1.87, 0.87) | -0.72  (-1.77, 0.58) | 0.20  (-0.87, 1.12) | 0.25 | -0.41  (-1.61, 0.92) | -0.39  (-1.60, 0.96) | -0.01  (-0.97, 0.98) | 0.81 |
| **DV (cm^3^)** | -2.66  (-9.90, 4.10) | -3.03  (-10.90, 3.13) | 0.62  (-6.47, 8.57) | 0.23 | -1.85  (-8.27, 4.43) | -2.22  (-8.61, 4.47) | -0.06  (-5.61, 5.87) | 0.79 |

**Table S4: Median (IQR) Changes and Differences between Sides Stratified on Tumor Size for those Age 55+**

|  | **IPSI Change** | **Contra Change** | **Difference** | **P-Value** |
| --- | --- | --- | --- | --- |
| **Tumors ≤2 cm (N=605)** | | | | |
| **VPD (%)** | -0.14  (-0.92, 0.66) | -0.23  (-1.04, 0.44) | 0.08  (-0.63, 0.82) | 0.06 |
| **DV (cm^3^)** | -1.81  (-7.71, 3.86) | -2.09  (-8.32, 3.06) | 0.22  (-5.08, 7.43) | 0.12 |
|  |  |  |  |  |
| **Tumors 2-5 cm (N=161)** | | | | |
| **VPD (%)** | -0.16  (-1.33, 0.77) | -0.42  (-1.59, 0.19) | 0.38  (-0.59, 1.44) | <.001 |
| **DV (cm^3^)** | -0.30  (-7.12, 6.25) | -1.95  (-11.14, 2.81) | 3.58  (-4.76, 11.85) | <.001 |
|  |  |  |  |  |
| **Tumors >5 cm (N=21)** | | | | |
| **VPD (%)** | 0.24  (-0.24, 1.93) | -0.30  (-0.92, 0.23) | 1.09  (0.01, 1.84) | 0.005 |
| **DV (cm^3^)** | 11.43  (-0.83, 19.88) | -2.60  (-6.54, -0.63) | 11.25  (1.45, 24.56) | <.001 |
